# A Few Remarks on Riggs Disease—Being an Excerpt from Some Correspondence

**Published:** 1916-07

**Authors:** Jules J. Sarrazin

**Affiliations:** New Orleans


### A FEW REMARKS ON RIGGS DISEASE—BEING AN EXCERPT FROM SOME CORRESPONDENCE.

BY JULES J. SARRAZIN, D.D.S., NEW ORLEANS.

The dystrophic atrophy of the jaws is an old enemy of mine, which I have been taking into account whenever at all indicated, making use of systemic theraphy and hygiene to benefit such conditions, lately adding the cupping method to local massage as an adjuvent. Therefore, jumping to the "conclusion" in a paper, which I have seen in print, and taking the liberty to paraphrase those conclusions to agree with my study of the etiology and pathology, I would say:

There is a form of inflammation which results in an organized exudate that contracts and leads to atrophy: cirrhotic, fibroid or sclerotic inflammation are examples. It will often have been acute at first, for a comparatively short period of time.

The predisposing cause of suppurative Rigg's Disease is atrophy, resulting from dystrophia. Infection of such tissues produces atrophic inflammation. The determining cause is infection from bacterial films, salivary calculi, and all agencies which tend to retain septic matter in contact with gingival margins, such as irregularities, or bad dentistry.

Serumal calculi are formed by exudates from capillaries breaking down in the process of acute or atrophic inflammation, as one source, while disintegrating bone supplies the other; and the result of both attaches to cementum where suppuration has destroyed pericementum. Serumal calculi and infected cementum must both be removed. So much infected bone from a rib, or elsewhere. Cementum lacunae must not be opened to infection.

Resistance of tissues is to be obtained, first by improving the quality of blood and its circulation, and second by allowing enough time between operations, for the results of vaccination to develop. Of course, traumatism induced plays a part in the process of repair, but antibodies developed after each inoculation of tissues, in the process of operating pockets and curetting carious bone, are an important factor.

The chemico-traumatic sulphuric acid treatment is, I think, an error, except in locations where the operator can have no certainty of having surgically removed, absolutely every trace of infected material. The blood clot method yields more rapidly, and satisfactory results, provided that all infection has been removed. Hyperemia induced by instrumentation is sufficient. Even mixed with glycerin, sulphuric acid is vastly more destructive to tissue cells than iodine. The desideratum is to preserve as much periodontal tissue as possible.

Dr. Eugene S. Talbot's old formula (Iodo-Glycerole) iodine crystals with zinc Iodid and glycerin, is invaluable as a germicidal protection and tissue stimulant after the initial traumatism has subsided. Tissue cells are saved by protection against recurrent infection.—*American Dentist.*

